# Upregulation of miR-93 and inhibition of LIMK1 improve ventricular remodeling and alleviate cardiac dysfunction in rats with chronic heart failure by inhibiting RhoA/ROCK signaling pathway activation

**DOI:** 10.18632/aging.102272

**Published:** 2019-09-20

**Authors:** Qian Su, Peng Zhang, Dong Yu, Zhaodi Wu, Dandan Li, Fangfang Shen, Pengfei Liao, Guizhi Yin

**Affiliations:** 1 Cardiovascular Department, Minhang Hospital, Fudan University, Shanghai 201199, P.R. China

**Keywords:** microRNA-93, LIMK1, RhoA/ROCK pathway, chronic heart failure, ventricular remodeling

## Abstract

Objective: There are some researches about the role of microRNA (miRNA) in chronic heart failure (CHF) were performed, but the study about miR-93’s function in CHF is scarcely investigated. Thus, we determined to probe into the effects of miR-93 in rats with CHF by targeting LIMK1 through regulating RhoA/ROCK pathway.

Results: We found increased LIMK1 and decreased miR-93 in CHF rats, and up-regulation of miR-93 inhibited LIMK1, RhoA and ROCK1 expression in CHF rats. Up-regulation of miR-93 or inhibition of LIMK1 decreased oxidative stress, inflammatory factors, as well as apoptosis-related indicators in CHF rats. LIMK1 was confirmed as a direct target gene of miR-93.

Conclusion: Our study provides evidence that upregulated miR-93 and downregulated LIMK1 improve ventricular remodeling and reduce cardiac dysfunction in CHF rats by inhibiting RhoA/ROCK signaling pathway activation.

Methods: First, rat models of CHF were established by aortic coarctation, and the rats were injected with miR-93 mimics, LIMK1-siRNA or overexpressed-LIMK1. Then expression of miR-93, LIMK1, RhoA, and ROCK1 expression in myocardial tissues were detected, after which indices of cardiac ultrasound, hemodynamics, and oxidative stress, inflammatory factors, apoptosis-related indicators were detected via a series of assays. Finally, the targeting relationship of miR-93 and LIMK1 was verified.

## INTRODUCTION

Chronic heart failure (CHF) is a conventional, severe, and disabling but treatable syndrome, and the pharmacological therapy for CHF includes a mixture of neurohormonal antagonists covering an angiotensin converting enzyme inhibitor, angiotensin receptor blocker, beta-blocker, and/or aldosterone antagonist [1]. CHF remains the final ordinary endpoint of the most of cardiac conditions, anaemia of unascertained origin is frequently presented in CHF patients and leaves an even inferior prognosis [2]. Various factors are related to increased mortality and morbidity in patients with CHF, such as demographic, clinical, and laboratory variables, and its syndrome involves the concurrent activation of multiple neurohormonal systems [3]. Patients with CHF have characteristics of autonomic dysfunction characterized by excessive sympathetic activation and accompanying parasympathetic withdrawal [4]. Patients with CHF with a preserved ejection fraction have descending ventricular relaxation, disordered active relaxation, and/or an increase in ventricular and arterial stiffness. Patients with a depressed ejection fraction have a disordered contraction pattern, most of them also have diastolic dysfunction [5]. At present, some microRNAs (miRNAs), such as miR-21, miR-378, and miR-940 are reported to be involved in CHF [6], but researches about function of miR-93 in CHF are hardly discussed, thus our study was aimed to explore an effective therapeutic mechanism concerning miR-93 for CHF treatment.

MiRNAs, a member of small noncoding RNAs family that target specific sequences of mRNAs to regulate gene expression, with more than 60% of mammalian mRNAs are target of miRNAs [7]. MiR-93, which derived from a paralogue (miR-106b-25) of miR-17-92 cluster, is reported to be up-regulated in many types of cancers [8], and has been reported to be a negative regulator of the immune response [9]. In some types of ischemic disease like myocardial infarction, stoke as well as peripheral arterial disease, miR-93 has been found to exhibit long-term protective roles via elevating angiogenesis [10]. Dickinson BA et al. have found that the circulating levels of miR-93 and other miRNAs are found in response to hypertension-induced heart failure [11]. LIM domain kinase 1 (LIMK1), a serine/threonine kinase, which controls actin polymerization by phosphorylation and inactivation of cofilin [12]. There is a association between LIMK1 and Alzheimer's disease (AD), which explains some neurological problems like impaired long-term memory, cerebellar ataxia, as well as impaired synaptic plasticity detected in AD [13]. Nevertheless, there is no study concentrated on the role of LIMK1 in CHF. Involved in cell proliferation and invasion, LIMK1 is primarily activated by Pak, Rho kinase (ROCK) and MRCK (downstream effectors of the Rho family of small GTPases). LIMK1 and its upstream signaling factors could work as valuable targets for anti-cancer metastasis therapies [14]. ROCK is a serine/threonine kinase and a downstream target of the small GTPase Rho, and the RhoA/ROCK pathway is involved in many neuronal functions such as migration, dendrite development, and axonal extension [15]. A study has revealed that RhoA/ROCK pathway could mediate immune responses in liver ischemia and reperfusion injury [16]. Luo et al. have stated that the cardioprotection of myocardial ischemia-reperfusion injury may be related to the inhibition of RhoA/ROCK pathway [17]. Another study has revealed that chronic sildenafil treatment could attenuate left ventricular dysfunction of ischemic heart failure, and this cardioprotection is associated with the suppression of the RhoA/Rho-kinase pathway [18]. But, the mechanisms of miR-93 mediating CHF has not been explored yet. Therefore, this research was determined to verify the mechanism of miR-93 along with LIMK1 and RhoA/ROCK pathway involvement in the procedure of CHF.

## RESULTS

### Up-regulation of miR-93 inhibits LIMK1, RhoA and ROCK1 expression in myocardial tissues

The miR-93, LIMK1, RhoA and ROCK1 expression in myocardial tissues of each group was detected by RT-qPCR and western blot assay. The results showed that the expression of miR-93 in the CHF group was obviously lower than that in the sham group, and the expression levels of LIMK1, RhoA and ROCK1 were dramatically increased (all *P* < 0.05). In contrast to the mimics-NC group, the expression level of miR-93 was obviously increased in the miR-93 mimics group, and the expression levels of LIMK1, RhoA and ROCK1 were obviously decreased (all *P* < 0.05). In contrast to the siRNA-NC group, the expression level of miR-93 in the LIMK1-siRNA group did not change dramatically (*P* > 0.05), and the expression levels of LIMK1, RhoA and ROCK1 were obviously decreased (all *P* < 0.05). No significant differences were found in expression levels of LIMK1, RhoA and ROCK1 among CHF group, mimics-NC group, siRNA-NC group and miR-93 mimics + OE-LIMK1 group (all *P* > 0.05).

**Figure 1:**
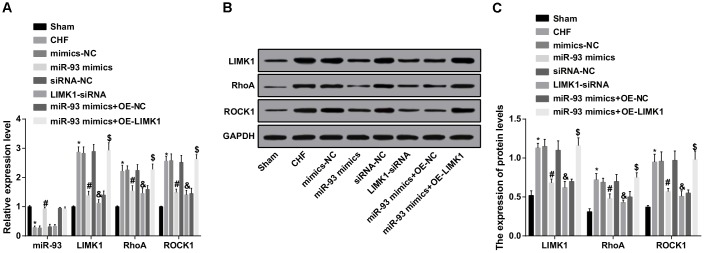
**LIMK1, RhoA and ROCK1 mRNA expression was inhibited by up-regulation of miR-93.** (**A**) miR-93, LIMK1, RhoA and ROCK1 expression levels in myocardial tissues of each group by RT-qPCR; (**B**) Protein bands of LIMK1, RhoA and ROCK1 in myocardial tissues; (**C**) LIMK1, RhoA and ROCK1 protein expression in myocardial tissues of all group by western blot assay; * *P* < 0.05 vs. the sham group; #*P* < 0.05 vs. the mimics-NC group; & *P* < 0.05 vs. the siRNA-NC group; $ *P* < 0.05 vs. the miR-93 mimics + OE-NC group; N = 8; the data is expressed as mean ± standard deviation; the data are analyzed by using one-way ANOVA, and LSD-t method was used for pairwise comparison after ANOVA.

To further investigate the effect of miR-93 targeting LIMK1 gene on the expression of miR-93, LIMK1, RhoA and ROCK1 in myocardial tissues of rats with CHF, RT-qPCR and western blot assay were involved and the results showed that in contrast to the miR-93 mimics + OE-NC group, no obvious change was found in the expression of miR-93 in the miR-93 mimics + OE-LIMK1 group (*P* > 0.05), and the expression levels of LIMK1, RhoA and ROCK1 were obviously increased (all *P* < 0.05; Figure 1A–1C).

### Up-regulation of miR-93 or downregulation of LIMK1 decreases LVIDd, LVIDs, LVEDP, LVMI, RVMI and increases LVEF, LVFS, +dp/dt max and -dp/dt max

The results of echocardiography, hemodynamics and ventricular mass index of the rats in all group showed that the levels of LVIDd, LVIDs, LVEDP, LVMI and RVMI in the CHF group were dramatically higher than their expression levels in the sham group, and LVEF, LVFS, +dp/dt max and -dp/dt max levels were obviously reduced (all *P* < 0.05). In contrast to the mimics-NC group, the levels of LVIDd, LVIDs, LVEDP, LVMI, and RVMI were dramatically decreased in the miR-93 mimics group, and the levels of LVEF, LVFS, +dp/dt max, and -dp/dt max were obviously increased (all *P* < 0.05). In contrast to the siRNA-NC group, LVIDd, LVIDs, LVEDP, LVMI, and RVMI levels were obviously decreased in the LIMK1-siRNA group, and LVEF, LVFS, +dp/dt max and -dp/dt max levels were dramatically increased (all *P* < 0.05). LVIDd, LVIDs, LVEDP, LVEF, LVFS, +dp/dt max, -dp/dt max, LVMI, and RVMI levels in the CHF group, mimics-NC group, siRNA-NC group, and miR-93 mimics+ OE-LIMK1 group showed no obvious difference (all *P* > 0.05).

**Figure 2:**
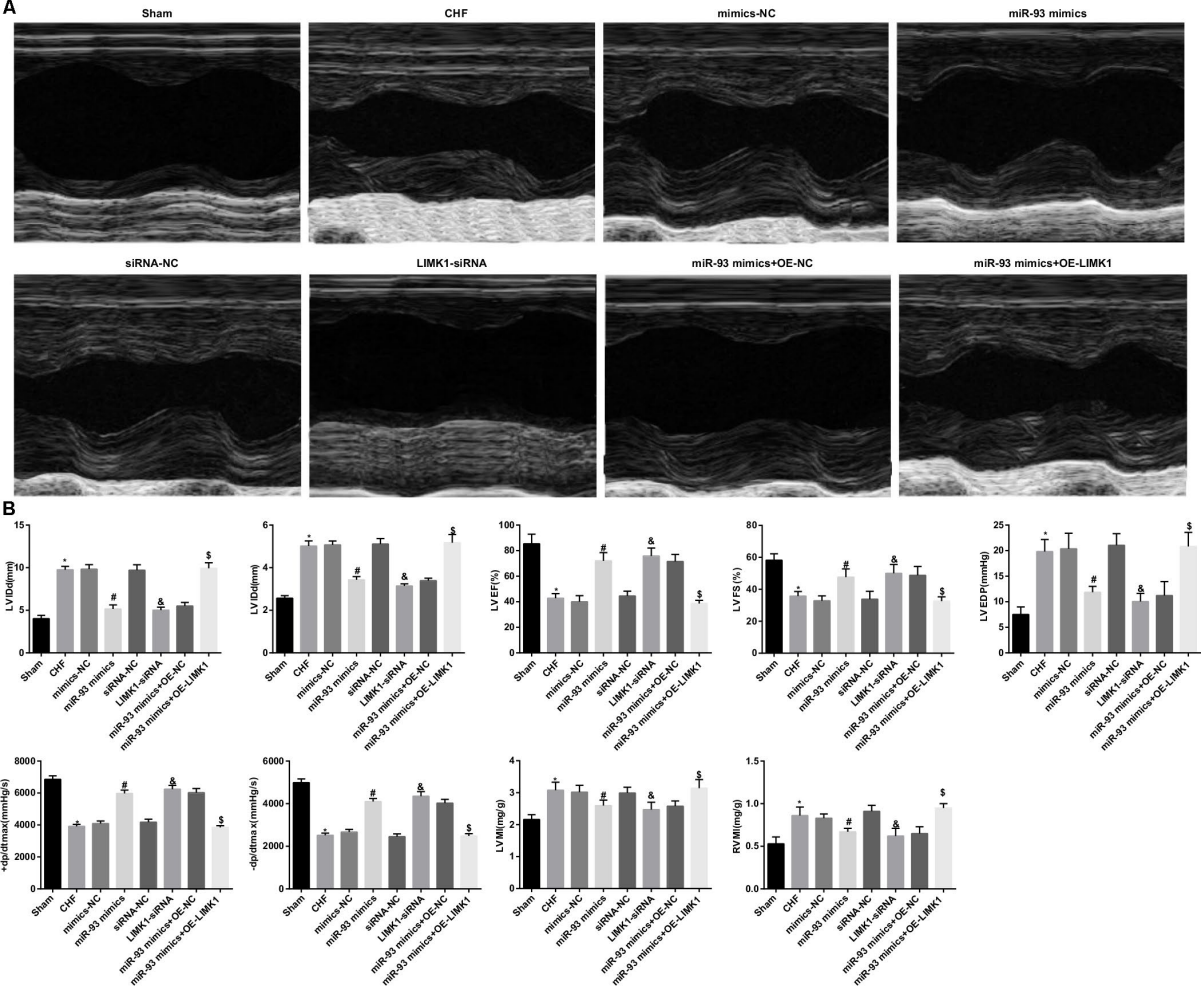
**LVIDd, LVIDs, LVEDP, LVMI and RVMI levels were decreased and LVEF, LVFS, +dp/dt max and -dp/dt max were increased by up-regulation of miR-93 or downregulation of LIMK1.** (**A**) Comparison of echocardiograms of rats in each group; (**B**) Comparison of LVIDd, LVIDs, LVEF, LVFS, LVEDP, +dp/dt max, -dp/dt max, LVMI and RVMI in each group; * *P* < 0.05 vs. the sham group; # *P* < 0.05 vs. the mimics-NC group; &*P* < 0.05 vs. the siRNA-NC group; $*P* < 0.05 vs. the miR-93 mimics + OE-NC group; N = 8, the data were expressed as mean ± standard deviation; data analysis was performed by one-way ANOVA, and LSD-t method was used for pairwise comparison after ANOVA.

To further investigate the effect of miR-93 targeting LIMK1 gene on echocardiography, hemodynamics and ventricular mass index in rats with CHF, the results of which showed that the levels of LVIDd, LVIDs, LVEDP, LVMI and RVMI in the miR-93 mimics + OE-LIMK1 group were significantly higher than those in the miR-93 mimics + OE-NC group, LVEF and LVFS, +dp/dt max and -dp/dt max levels were dramatically reduced (all *P* < 0.05; Figure 2A–2B).

### Up-regulation of miR-93 or downregulation of LIMK1 reduces BNP, cTnI, AngII and NE levels in plasma of rats

The changes of plasma BNP, cTnI, AngII and NE in the rats of each group were detected by ELISA. The results revealed that the levels of BNP, cTnI, AngII and NE in the CHF group were dramatically higher than those in the sham group (all *P* < 0.05). In contrast to the mimics-NC group, the levels of BNP, cTnI, AngII and NE in the miR-93 mimics group were obviously declined (all *P* < 0.05). In contrast to the siRNA-NC group, the plasma levels of BNP, cTnI, AngII, and NE were obviously lower than those in the LIMK1-siRNA group (all *P* < 0.05). No significant differences were found in plasma BNP, cTnI, AngII and NE levels among the CHF group, mimics-NC group, siRNA-NC group and the miR-93 mimics + OE-LIMK1 group (all *P* > 0.05).

**Figure 3:**
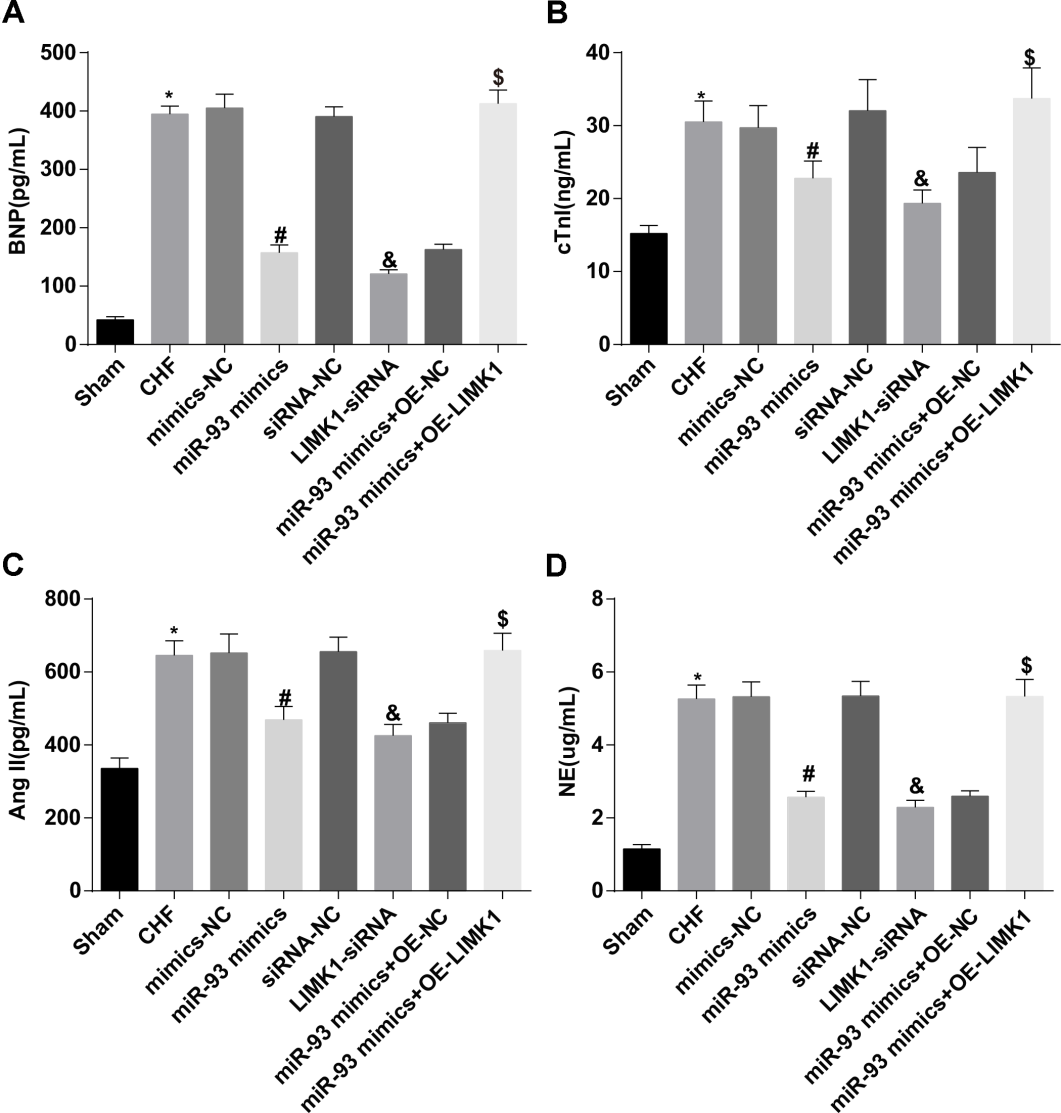
**BNP, cTnI, AngII and NE levels were reduced by up-regulation of miR-93 or downregulation of LIMK1.** (**A**) Comparison of plasma BNP levels in each group of rats; (**B**) Comparison of plasma cTnI levels in each group; (**C**) Comparison of plasma AngII levels in each group; (**D**) Comparison of NE levels in plasma of each group; *P < 0.05 vs. the sham group; #*P* < 0.05 vs. the mimics-NC group; & *P* < 0.05 vs. the siRNA-NC group; $P < 0.05 vs. the miR-93 mimics + OE-NC group; N = 8, the data were showed as mean ± standard deviation; data analysis was performed by one-way ANOVA, and LSD-t method was used for pairwise comparison after ANOVA.

To further investigate the effect of miR-93 targeting LIMK1 gene on plasma BNP, cTnI, AngII and NE in rats with CHF, the results showed that in contrast to the miR-93 mimics + OE-NC group, the levels of BNP, cTnI, AngII and NE in the plasma of the miR-93 mimics + OE-LIMK1 group were obviously increased (all *P* < 0.05; Figure 3A–3D).

### Up-regulation of miR-93 or downregulation of LIMK1 inhibits TNF-α, IL-1β and IL-6 levels in plasma and myocardial tissues of rats

The levels of TNF-α, IL-1β and IL-6 in plasma and myocardial tissues were measured by ELISA and western blot analysis. The results indicated that the levels of TNF-α, IL-1β and IL-6 in plasma and myocardial tissues in the CHF group were dramatically higher than those in the sham group (all *P* < 0.05). In contrast to the mimics-NC group, the levels of TNF-α, IL-1β, and IL-6 in the miR-93 mimics group were obviously decreased (all *P* < 0.05). In contrast to the siRNA-NC group, the levels of TNF-α, IL-1β, and IL-6 in the LIMK1-siRNA group were obviously declined (all *P* < 0.05). No significant differences were found in levels of TNF-α, IL-1β and IL-6 in plasma and myocardial tissues among the CHF group, mimics-NC group, siRNA-NC group and miR-93 mimics+ OE-LIMK1 group (all *P* > 0.05).

**Figure 4:**
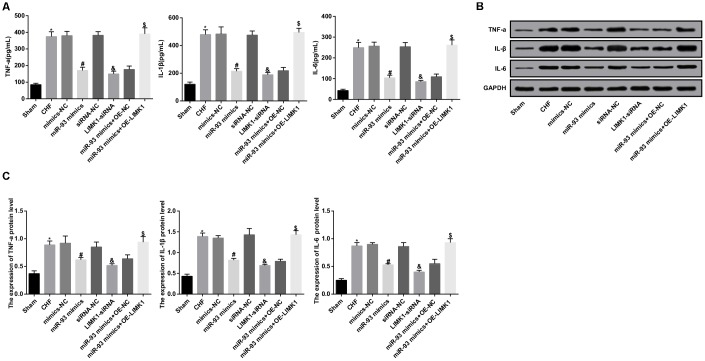
**TNF-α, IL-1β, IL-6 levels were inhibited by up-regulation of miR-93 or downregulation of LIMK1.** (**A**) Comparison of TNF-α, IL-1β, IL-6 levels in plasma of each group rats; (**B**) Protein bands of TNF-α, IL-1β, IL-6 in myocardial tissue of each group; (**C**) The protein expression of TNF-α, IL-1β, and IL-6 in myocardial tissue of each group;*P < 0.05 vs. the sham group; #*P* < 0.05 vs. the mimics-NC group; & *P* < 0.05 vs. the siRNA-NC group; $ *P* < 0.05 vs. the miR-93 mimics + OE-NC group; N = 8, the data were showed as mean ± standard deviation; data analysis was performed by one-way ANOVA, and LSD-t method was used for pairwise comparison after ANOVA.

To further investigate the effect of miR-93 targeting LIMK1 gene on TNF-α, IL-1β, and IL-6 in plasma and myocardial tissues in rats with CHF, the results showed that in contrast to the miR-93 mimics + OE-NC group, the levels of TNF-α, IL-1β and IL-6 in the the miR-93 mimics + OE-LIMK1 group were obviously increased (all *P* < 0.05; Figure 4A–4C).

### Up-regulation of miR-93 or downregulation of LIMK1 inhibits MDA levels and promotes SOD and T-AOC levels in plasma of rats

The findings of oxidative stress test showed that in contrast to the sham group, the MDA level in the plasma of the CHF group was obviously increased, while SOD and T-AOC levels were dramatically decreased (all *P* < 0.05). In contrast to the mimics-NC group, the MDA level in the plasma of the miR-93 mimics group was obviously decreased, and SOD and T-AOC levels were obviously increased (all *P* < 0.05). In contrast to the siRNA-NC group, MDA level in the plasma of the LIMK1-siRNA group was obviously decreased, and SOD and T-AOC levels were dramatically increased (all *P* < 0.05). No significant differences were found in plasma SOD, MDA and T-AOC levels among the CHF group, mimics-NC group, siRNA-NC group and the miR-93 mimics + OE-LIMK1 group (all *P* > 0.05).

**Figure 5:**
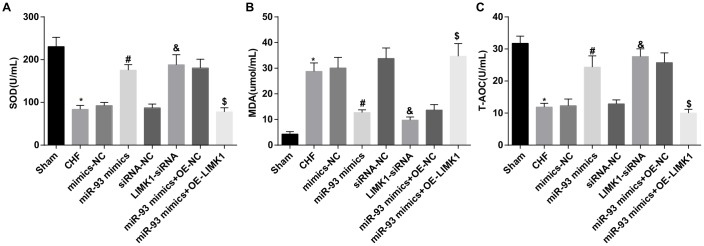
**MDA level was promoted while SOD and T-AOC levels were inhibited by up-regulation of miR-93 or downregulation of LIMK1.** (**A**) Comparison of plasma SOD content in each group; (**B**) Comparison of plasma MDA content in each group; (**C**) Comparison of plasma T-AOC content in each group; *P < 0.05 vs. the sham group; #*P* < 0.05 vs. the mimics-NC group; & *P* < 0.05 vs. the siRNA-NC group; $*P* < 0.05 vs. the miR-93 mimics + OE-NC group; N = 8, the data were showed as mean ± standard deviation; data analysis was performed by one-way ANOVA, and LSD-t method was used for pairwise comparison after ANOVA

In order to further investigate the effect of miR-93 targeting LIMK1 gene on plasma oxidative stress in rats with CHF, the results showed that MDA levels were obviously increased, and SOD and T-AOC levels were dramatically decreased in the miR-93 mimics + OE-LIMK1 group relative to that in the miR-93 mimics +OE-NC group (all *P* < 0.05; Figure 5A–5C).

### Up-regulation of miR-93 or downregulation of LIMK1 inhibits LDH, AST, CK and CK-MB levels in plasma of rats

The results of myocardial enzyme test showed that LDH, AST, CK and CK-MB levels in the CHF group were dramatically higher than those in the sham group (all *P* < 0.05). In contrast to the mimics-NC group, LDH, AST, CK, and CK-MB levels in the miR-93 mimics group were obviously reduced (all *P* < 0.05). In contrast to the siRNA-NC group, LDH, AST, CK, and CK-MB levels in the LIMK1-siRNA group were obviously declined (all *P* < 0.05). No significant differences were found in plasma levels of LDH, AST, CK and CK-MB among the CHF group, mimics-NC group, siRNA-NC group and the miR-93 mimics + OE-LIMK1 group (all *P* > 0.05).

**Figure 6:**
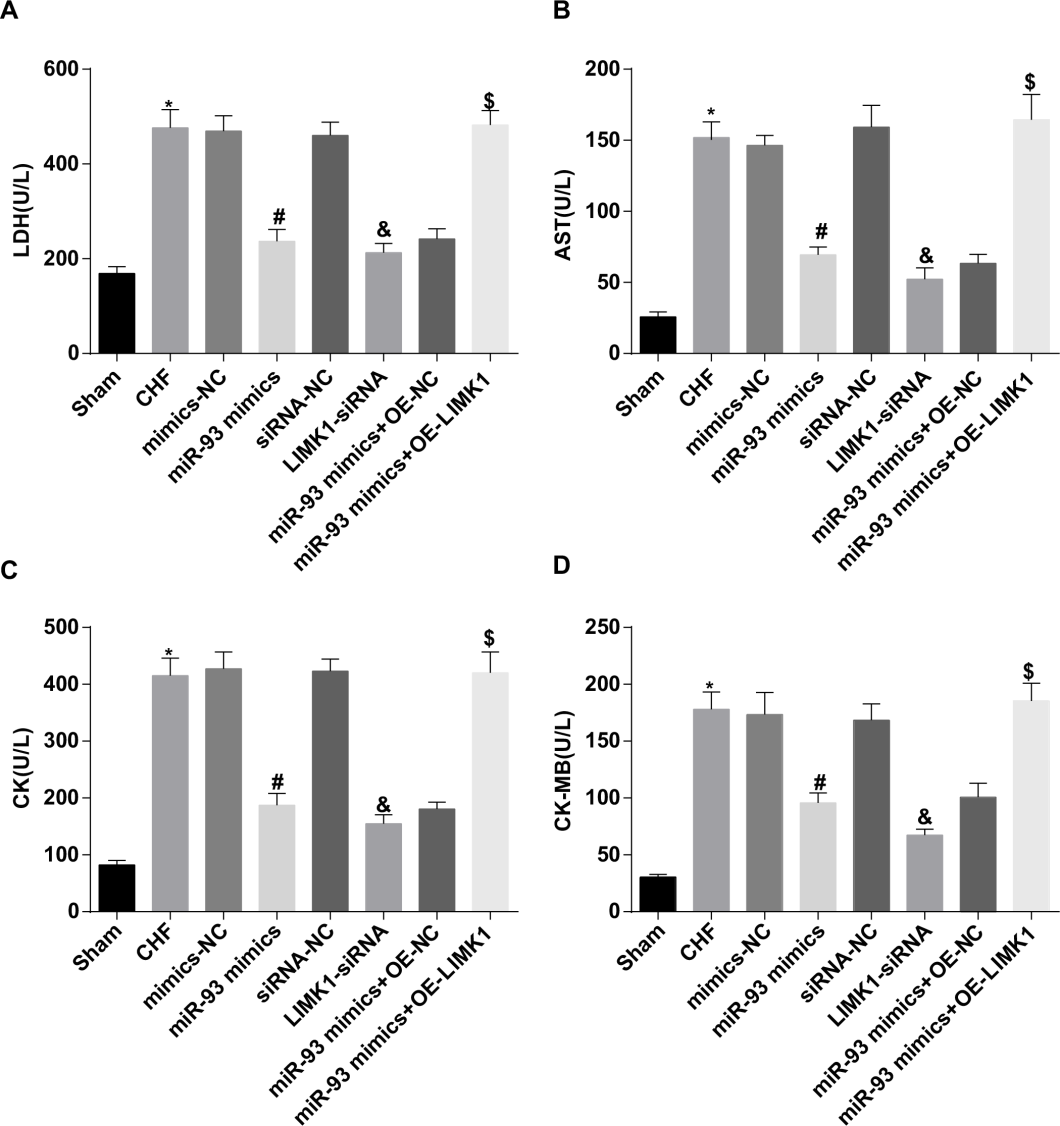
**LDH, AST, CK and CK-MB levels were inhibited by up-regulation of miR-93 or downregulation of LIMK1.** (**A**) Comparison of plasma LDH content in each group of rats; (**B**) Comparison of plasma AST content in each group of rats; (**C**) Comparison of plasma CK content in each group of rats; (**D**) Comparison of CK-MB content in plasma of each group of rats; *P < 0.05 vs. the sham group; #*P* < 0.05 vs. the mimics-NC group; & *P* < 0.05 vs. the siRNA-NC group; $*P* < 0.05 vs. the miR-93 mimics + OE-NC group; N = 8, the data were showed as mean ± standard deviation; data analysis was performed by one-way ANOVA, and LSD-t method was used for pairwise comparison after ANOVA.

To further investigate the effect of miR-93 targeting LIMK1 gene on plasma myocardial enzymes in rats with CHF, the results showed that in contrast to the miR-93 mimics + OE-NC group, LDH, AST, CK, and CK-MB levels in rat plasma were obviously increased in the miR-93 mimics + OE-LIMK1 group (all *P* < 0.05; Figure 6A–6D).

### Up-regulation of miR-93 or downregulation of LIMK1 inhibits myocardial histopathological changes in rats

**Figure 7:**
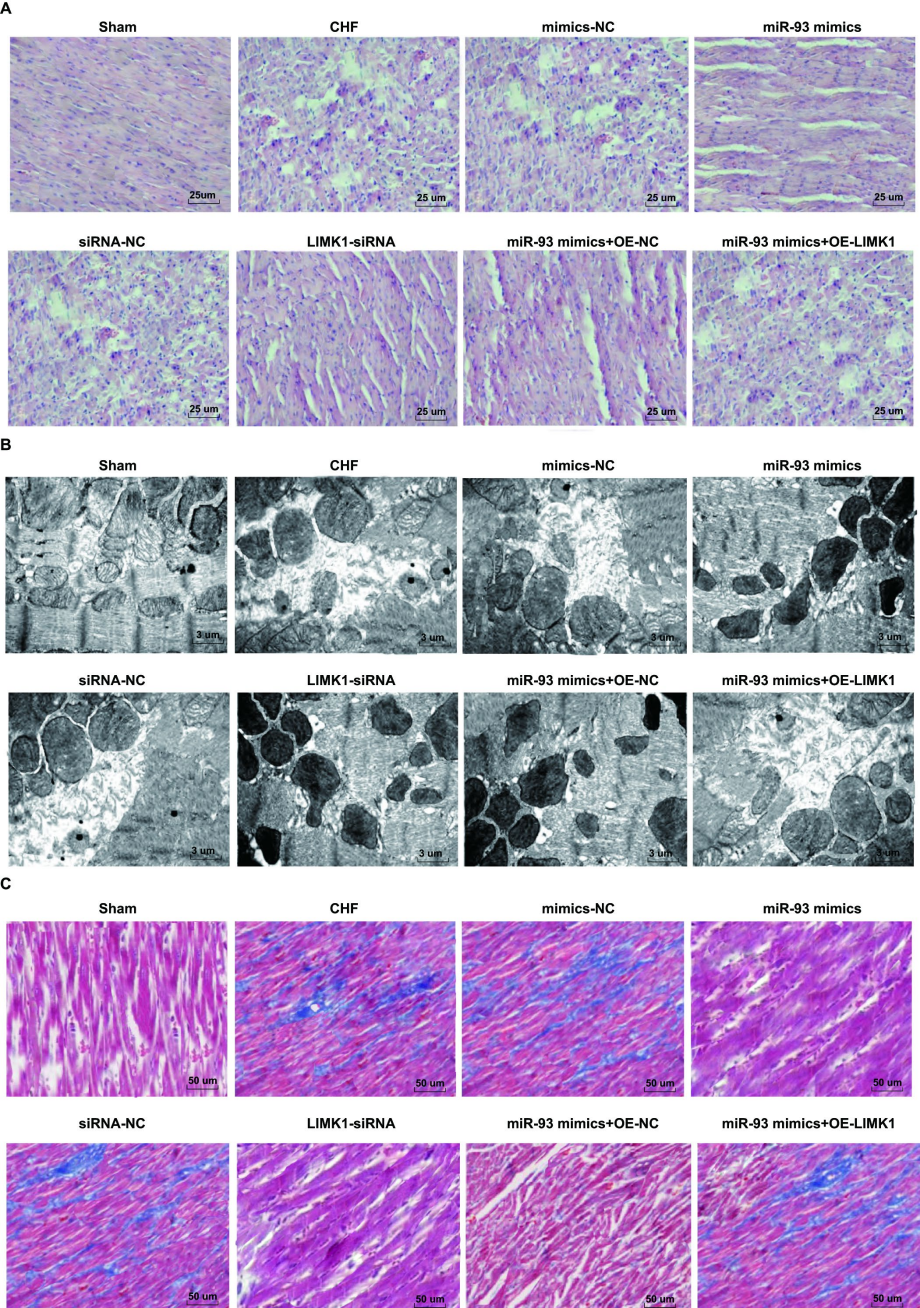
**Myocardial histopathological changes in rats were inhibited by up-regulation of miR-93 or downregulation of LIMK1.** (**A**) HE staining of myocardial tissues in each group (× 400, scale bar 25 μm); (**B**) Ultrastructure of cardiomyocytes in each group (× 3000, scale bar 3 μm); (**C**) Masson staining of myocardial tissues in each group (× 200, scale bar 50 μm).

The results of HE staining showed that the number, size and shape of rats’ cardiomyocytes were normal in the sham group, the staining was uniform and clear, the myocardial muscle fibers were arranged neatly, and no abnormal lesions such as deformation, hyperplasia and necrosis were observed. In the CHF group, mimics-NC group, siRNA-NC group, and miR-93 mimics + OE- LIMK1 group, rats’ cardiomyocytes were found swelling, necrosis, necrotic area myocardial fibrosis with inflammatory cell infiltration, myocardial cell gap increased with edema, nuclear staining, cytoplasmic vacuolization, myocardial fibers were broken and dissolved, and arranged disorderly. The cardiomyocytes of rats in the miR-93 mimics group, LIMK1-siRNA group, miR-93 mimics + OE-NC group were uniformly colored, the number was basically normal, the muscle fibers were arranged neatly, and the degree of fibrosis, the degree of restructuring and the degree of necrosis of cardiomyocytes were relatively mild (Figure 7A).

TEM showed that the mitochondria of the sham group were uniform, the structure was intact, the mitochondria were clear and intact, and the muscle fibers were arranged neatly. In the CHF group, mimics-NC group, siRNA-NC group, miR-93 mimics + OE-LIMK1 group, the mitochondria of cardiomyocytes were different in shape and size, and there were swelling, vacuolization, partial mitochondrial lysis and rupture, muscle fiber arrangement disorder, or even rupture cells. In the miR-93 mimics group, LIMK1-siRNA group, miR-93 mimics+ OE-NC group, the mitochondrial swelling of cardiomyocytes was not obvious with partial deformation, mitochondrial crest arrangement was slightly disordered, muscle fibers were arranged neatly, and there was mild dissolution (Figure 7B).

Masson staining showed that the collagen fibers were blue and the cardiomyocytes were red. In the sham group, the myocardial fibers were arranged neatly and uniformly, without collagen. In the CHF group, mimics-NC group, siRNA-NC group, and miR-93 mimics + OE-LIMK1 group, there were obvious myocardial fibrosis, and a large amount of cardiomyocytes were necrotic, collagen composition was increased significantly, and the degree of fibrosis was obvious. In the miR-93 mimics group, LIMK1-siRNA group, miR-93 mimics + OE-NC group, cardiomyocytes were increased and arranged neatly, and the collagen fibers decreased obviously (Figure 7C).

### Up-regulation of miR-93 or downregulation of LIMK1 inhibits Cleaved caspase-3, Bax levels and promotes Bcl-2 level in cardiomyocytes of rats

The results of TUNEL staining showed that the cardiomyocyte apoptosis index (AI) of the CHF group was obviously higher than that in the sham group (*P* < 0.05). In contrast to the mimics-NC group, the AI of cardiomyocytes in the miR-93 mimics group was obviously reduced (*P* < 0.05). In contrast to the siRNA-NC group, the AI of cardiomyocytes in the LIMK1-siRNA group was dramatically declined (*P* < 0.05). No significant differences were found in AI of cardiomyocytes among the CHF group, mimics-NC group, siRNA-NC group and the miR-93 mimics + OE-LIMK1 group (*P* > 0.05).

**Figure 8:**
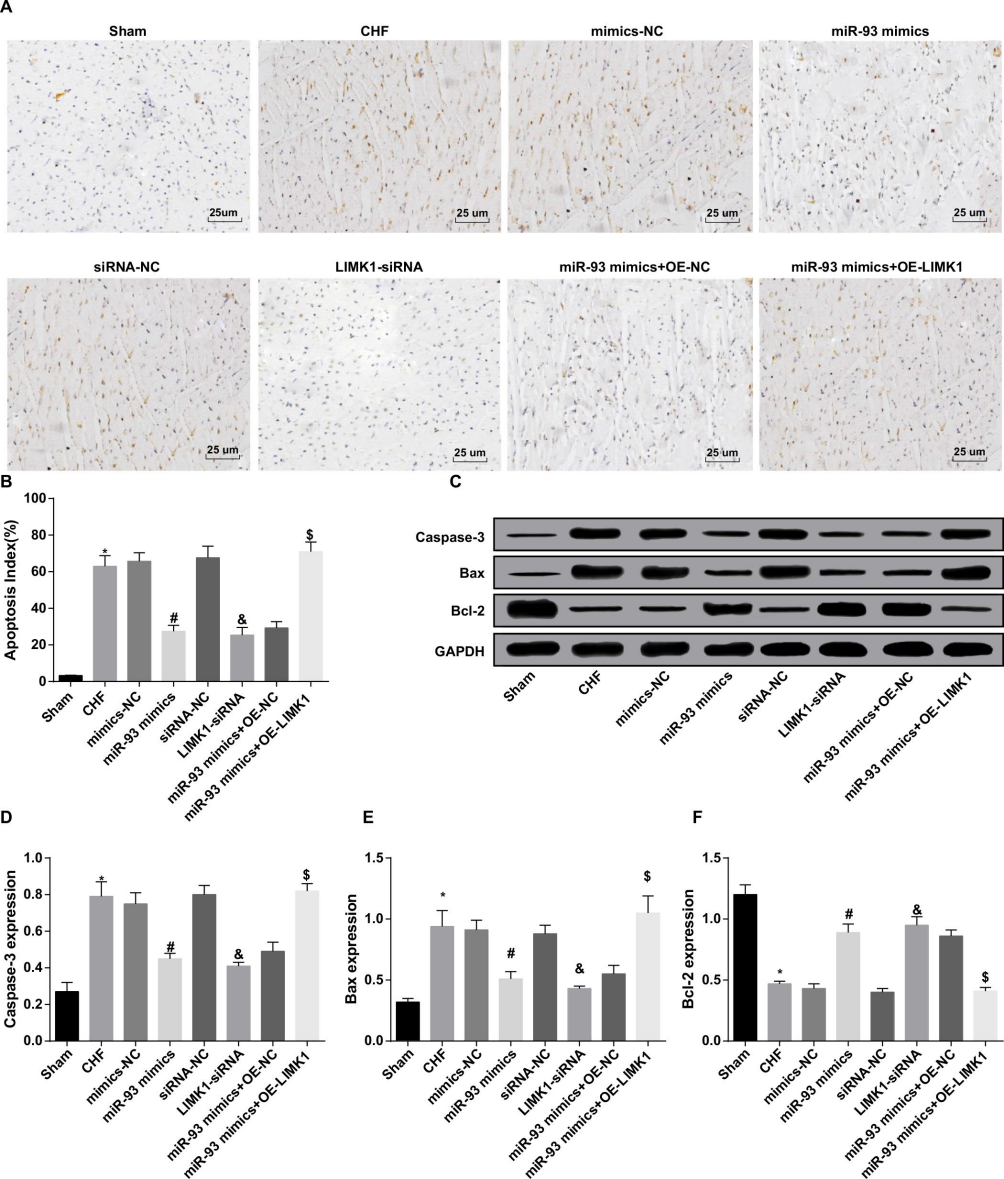
**Cleaved caspase-3 and Bax levels were inhibited and Bcl-2 level was promoted by up-regulation of miR-93 or downregulation of LIMK1.** (**A**) TUNEL staining was used to observe the apoptosis of cardiomyocytes in each group (× 400, scale bar 25 μm); (**B**) Apoptosis index of cardiomyocytes in each group; (**C**) Protein bands of Cleaved caspase-3, Bax, and Bcl-2 protein in myocardial tissues of each group; (**D**) Protein expression of Cleaved caspase-3 in myocardial tissue of each group; (**E**) Protein expression of Bax in myocardial tissues of rats in each group; (**F**) Protein expression of Bcl-2 in myocardial tissues of each group; *P < 0.05 vs. the sham group; #*P* < 0.05 vs. the mimics-NC group; & *P* < 0.05 vs. the siRNA-NC group; $*P* < 0.05 vs. the miR-93 mimics + OE-NC group; N = 8, the data were showed as mean ± standard deviation; data analysis was performed by one-way ANOVA, and LSD-t method was used for pairwise comparison after ANOVA.

To further investigate the effect of miR-93 targeting LIMK1 gene on cardiomyocyte AI in rats with CHF, the results showed that in contrast to the miR-93 mimics + OE-NC group, the cardiomyocyte AI in the miR-93 mimics + OE-LIMK1 group was obviously increased (*P* < 0.05; Figure 8A–8B).

The results from western blot assay suggested that in contrast to the sham group, Cleaved caspase-3 and Bax expression levels in the CHF group were obviously increased, and Bcl-2 protein expression was dramatically decreased (all *P* < 0.05). In contrast to the mimics-NC group, Cleaved caspase-3 and Bax expression levels in the miR-93 mimics group were obviously decreased, and Bcl-2 protein expression was dramatically increased (all *P* < 0.05). In contrast to the siRNA-NC group, Cleaved caspase-3 and Bax protein expression levels in the LIMK1-siRNA group were obviously decreased, and Bcl-2 protein expression level was dramatically elevated (all *P* < 0.05). No significant differences were found in Cleaved caspase-3, Bax and Bcl-2 expression levels among the CHF group, mimics-NC group, siRNA-NC group and miR-93 mimics + OE-LIMK1 group (all *P* > 0.05).

To further investigate the effect of miR-93 targeting LIMK1 gene on apoptosis-related proteins expression in CHF rats, the results showed that in contrast to the miR-93 mimics + OE-NC group, Cleaved caspase-3 and Bax protein expression levels in the miR-93 mimics + OE-LIMK1 group were obviously increased, and Bcl-2 protein expression level was dramatically decreased (all *P* < 0.05; Figure 8C–8F).

### LIMK1 is a direct target gene of miR-93

**Figure 9:**
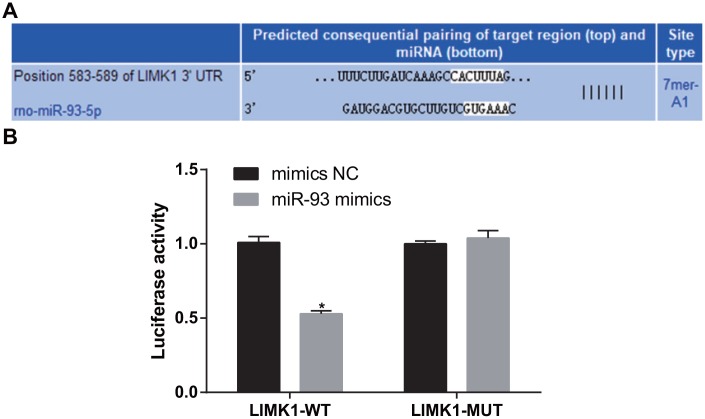
**LIMK1 was verified as a direct target gene of miR-93.** (**A**) Online software predicted the binding site of miR-93 and LIMK1; (**B**) Luciferase activity assay verified the targeting relationship between miR-93 and LIMK1; *P < 0.05 vs. the mimics-NC group.

The bioinformatics software http://www.targetscan.org predicted that miR-93 has a targeting relationship with LIMK1 (Figure 9A). The 293T cells were co-transfected with LIMK1-3'UTR-WT plasmid and miR-93 mimics. The results showed that in contrast to the LIMK1-3'UTR-WT + mimics NC group, the luciferase activity was obviously reduced in the LIMK1-3'UTR-WT + miR-93 mimics group (*P* < 0.05). In contrast to the LIMK1-3'UTR-MUT + mimics NC, no significant difference was found in luciferase activity in the LIMK1-3'UTR-MUT + miR-93 mimics group (*P* > 0.05; Figure 9B).

## DISCUSSION

Heart failure is a complicated clinical syndrome with a poor prognosis, and this syndrome is progressive and characterized by inferior life quality [19]. CHF caused by left ventricular systolic dysfunction is related to great morbidity, and then extensive use of evidence-based drugs and devices over the last decade have made significant improvements in CHF patients’ survival [20]. In the present study, we were determined to focus on the role of miR-93 in rats with CHF. We have found that up-regulation of miR-93 and inhibition of LIMK1 improve ventricular remodeling and reduce cardiac dysfunction in rats with CHF by inhibiting RhoA/ROCK signaling pathway activation.

A major new finding of this study is that up-regulation of miR-93 inhibits LIMK1, RhoA and ROCK1 expression in CHF rats. Similar to our results, some articles have discussed the relationship between other miRNAs with LIMK1. Likewise, a recent research discovered that miR-143 could affect LIMK1 expression in non-small cell lung cancer (NSCLC) tissues inversely [21]. Besides, as reported in a study, it was found that LIMK1 was affected negatively by miR-27b in NSCLC patients [22]. As for the associations of RhoA and ROCK1 with other miRNAs, another study has demonstrated that ROCK1 protein expression was greatly inhibited by miR-145 in the human glioma cell lines [23], which is in line with our study. Moreover, an earlier research proposed that ROCK1 was also inversely silenced by miR-145 in osteosarcoma cell tissues [24]. In this study, LIMK1 is also verified as a direct target gene of miR-93, which is also in accord with that LIMK1 is a target gene of miR-20a that may mediate the suppressive effects on growth and invasion of thyroid cancer cells [25]. However, there is no article concentrated on the targeting relationship between LIMK1 and miR-93, which is a new finding in this present study. Furthermore, in this present study, we found increased LIMK1 and decreased miR-93 in CHF rats. A study has suggested that miR-93 expression was lowly expressed in both mice and cellular models of cardiac hypertrophy [26], which is in line with the results of our study. LIMK1 levels are demonstrated to be increased in some human cancers, and upregulation of LIMK1 leads to tumor progression in prostate and breast cancer cells [27]. Nevertheless, the expression of LIMK1 in CFH or other cardiac diseases are scarcely explored. Meanwhile, we discovered that upregulation of miR-93 and inhibition of LIMK1 improve ventricular remodeling and alleviate cardiac dysfunction in rats with CHF. Likewise, miR-93 was verified to be downregulated in AngII-treated cardiomyocytes of cardiac hypertrophy [28], while the function of the inhibition of LIMK1 in CFH needs to be confirmed in future research.

Moreover, we discovered that LVIDd, LVIDs, LVEDP, LVMI, and RVMI were increased in CFH rats, while LVEF, LVFS, +dp/dt max and -dp/dt max were decreased. Meanwhile, a previous study has stated that LVIDs was increased, and +dp/dt max and -dp/dt max were depressed in diabetes patients [29]. BNP, cTnI, AngII and NE levels in CFH rats were found to be increased in our study, which was similar to the previous finding that preoperative NT-pro-BNP and cTNI levels were found to be significantly elevated in the individuals that experienced major adverse cardiac events than in those who did not [30]. Also, we found that MDA level was increased and SOD and T-AOC levels were decreased in CFH rats. On the foundation of our existing research results, we observed that the finding is closely related to a recent study highlighting that SOD activity in elderly coronary heart disease patients were dramatically lower than elderly non-coronary heart disease, while MDA level was greatly highly expressed [31]. Also, LDH, AST, CK and CK-MB levels were found to be enhanced in CHF rats of our study. In line with the results in our study, a study has highlighted that CK, LDH, AST, and CKMB were also elevated in coxsackievirus B3-induced myocardial tissues [32]. Still, we found that TNF-α, IL-1β and IL-6 levels were up-regulated in CHF rats. Similarly, a recent study has revealed that elevated TNF-α, IL-1β, and IL-6 mRNA expression in lipopolysaccharide-induced septic cardiac dysfunction mice was found [33]. Additionally, we have discovered that Cleaved caspase-3 and Bax expression levels were elevated and Bcl-2 expression level was declined in CHF rats, just as the previous research about that increased caspase-3 expression level, high Bax expression level and low Bcl-2 expression level were found in myocardial apoptosis cells along with pressure overload rat [34].

In conclusion, our study have revealed that upregulation of miR-93 and inhibition of LIMK1 improve ventricular remodeling and alleviate cardiac dysfunction in rats with CHF by inhibiting RhoA/ROCK signaling pathway activation. Our study suggests that miR-93 plays an vital role in the inhibition of cardiac dysfunction, which could be used as a new-generation biomarker for the prevention of CHF. Still, a more profound investigation of the mechanism is welcomed for scrupulously and logically work with a larger sample, as well as a better clinical application in therapeutic treatment for patients with CHF.

## MATERIALS AND METHODS

### Ethics statement

The study was approved by the Ethics Committee of Minhang Hospital, Fudan University, and informed written consent was obtained from all patients. All animal experiments were in accordance with the Guide for the Care and Use of Laboratory Animal by International Committees.

### Model preparation, animal grouping and treatment

Eighty Wistar rats (aged 7-8 weeks, weighted 230-280 g, purchased from Shanghai Kay Biological Technology Co., Ltd., Shanghai, China) were selected for our experiment. Breeding environment of rats was set: 12 h day and night alternation, clean grade, room temperature 20 - 25°C, humidity 55 - 60%, free to food and drinking water, as well as adaptable feeding for 1 w. Rat model of CHF was established by aortic coarctation [35]. After 1 w of adaptive feeding, the rats were shaved on the left chest and anesthetized by inhalation of 2.0% isoflurane. After tracheal intubation under a cold light source, the rats were fixed on a thermostatic surgical plate and connected to a ventilator. The iodophor disinfection operation area was used to open the thoracic layer between the second and third intercostals of the rats. After the aortic arch was separated, the 3-0 surgical suture was used to pass through the aortic arch, and the "L" needle with a diameter of 0.9 mm was placed in parallel. On the aortic arch, the "L" needle was gently pulled out. The chest cavity was sutured layer by layer, and the wound was disinfected. The penicillin (100,000 IU/d) was intramuscularly administered into rats 3 days after surgery, and the physiological condition of the rats was recorded. After 1 month, the model establishment was completed, and 8 rats died during the operation and 1 to 3 days after the operation, and 8 rats were replaced by the new rats for the modeling.

Sixty-four rats were successfully selected and divided into 8 groups: the sham group (except for the aortic coarctation, the rest of the operation was consistent with the CHF group), CHF group, mimics-negative control (NC) group (tail vein injection of the mixture of miR-93 mimics-NC, Entranster™-*in vivo* and glucose solution), miR-93 mimics group (tail vein injection of the mixture of miR-93 mimics, Entranster™-*in vivo* and glucose solution), siRNA-NC group (tail vein injection of the mixture of lowly-expressed LIMK1 NC, Entranster™-*in vivo* and glucose solution), LIMK1-siRNA group (tail vein injection of the mixture of lowly-expressed LIMK1, Entranster™-*in vivo* and glucose solution), miR-93 mimics + overexpression (OE)-NC group (tail vein injection of the mixture of miR-93 mimics and overexpressed LIMK1 NC, together with Entranster™-*in vivo* and glucose solution), and miR-93 mimics + OE-LIMK1 group (tail vein injection of the mixture of miR-93 mimics and overexpressed LIMK1, together with Entranster™-*in vivo* and glucose solution). Except for the CHF group and sham group, the rats in other groups were all cultured and adapted for 1 week. All rats were injected through tail vein with mimics-NC, miR-93 mimics, siRNA-NC, LIMK1-siRNA, miR-93 mimics and OE-NC, miR-93 mimics and OE-LIMK1 [36, 37], injection was performed every 3 days (4 weeks). And mimics-NC, MiR-93 mimics (The miRNA mimic synthesized by chemical method can simulate the high level expression of mature miRNA in cells in order to enhance the regulation of endogenous miRNA and carry out functional acquisition) and its mimics-NC, LIMK1 low expression vector (siRNA expression vector which can stably down-regulate gene expression) and its siRNA-NC, LIMK1 overexpression vector (overexpression vector which can stably up-regulate gene expression) and its OE-NC were prepared by Shanghai GenePharma Co. Ltd. (Shanghai, China). Entranster™-*in vivo* and glucose solution (animal *in vivo* transfection reagent, specially used for animals *in vivo* transfection, can directly transfer DNA or RNA into animal body) was purchased from Engreen Biosystem Co. Ltd. (Beijing, China).

### Cardiac ultrasound detection

The chest of the rat was placed in a supine position and the heart was ultrasonically examined with a Vivid 7 Dimension Color Doppler Ultrasound (General Electric Company, Boston, MA, USA) with a probe frequency of 13 MHz, a depth of 3.5 cm and a speed of 200 mm·s^-1^. After obtaining a satisfactory two-dimensional image of the left ventricle short axis of the sternum, the M-mode echocardiogram was obtained perpendicular to the interventricular septum and the posterior wall of the left ventricle at the papillary muscle level. The left ventricular end-diastolic diameter (LVIDd), left ventricular end-systolic diameter (LVIDs), left ventricular ejection fractions (LVEF), and left ventricular fractional shortening (LVFS) were measured.

### Hemodynamic testing

The rat peritoneal cavity was injected with 1% pentobarbital sodium, the neck tissue was bluntly separated, the rat carotid artery was exposed, and the telecentric end was ligated. Next, the proximal end was pulled with a silk thread to insert a 1.4F Millar catheter (Millar Instruments Inc., Houston, Texas, USA) to the rat carotid artery. The left ventricular end-diastolic pressure (LVEDP) and +dp/dt max and -dp/dt max were recorded when the Millar catheter entered the left ventricle. Whether or not the catheter entered the left ventricle chamber was determined based on the blood pressure waveform.

### Serum index testing

Rat abdominal aorta blood (5 mL) was taken, then centrifuged at 3000 r/min to obtain the supernatant. The enzyme-linked immunosorbent assay (ELISA) kit (Beckman Coulter Life Sciences, Brea, CA, USA) was used to determine the brain natriuretic peptide (BNP), cardiac troponin I (CTn-I), human angiotensin II (Ang II), norepinephrine (NE), tumor necrosis factor-α (TNF-α), interleukin-1β (IL-1β), and interleukin-6 (IL-6). Malondialdehyde (MDA) was detected by thiobarbituric acid method, the content of superoxide dismutase (SOD) was detected by xanthine oxidase method, total antioxidants (T-AOC) were detected by colorimetry. An automatic biochemical analyzer (Beckman Coulter Life Sciences, Brea, CA, USA) was used to detect the levels of lactic dehydrogenase (LDH), aspartate aminotransferase (AST), creatine kinase (CK) and creatine kinase isoenzyme (CK-MB). MDA, SOD, T-AOC, LDH, AST, CK, and CK-MB related reagents and kits were bought from Shenzhen Jingmei Biotechnology Co. Ltd. (Shenzhen, China).

### Measurement of ventricular index and collection of myocardial tissue specimens

All rats were euthanatized by exsanguination of the abdominal aorta, the large blood vessels of the heart were ligated, and a small opening was made in the right ventricle. The rats were perfused with pre-cooled 0.9% sodium chloride solution at 4°C. The ventricular chamber was flushed to a bloodless liquid, and the left and right ventricles were quickly weighed (including ventricular septum). Left ventricular mass index (LVMI, left ventricular mass/body mass) and right ventricular mass index (RVMI, right ventricular mass/body mass) were calculated. Myocardial tissues were cut into 2~3 mm wide, and some myocardial tissues were fixed in 4% paraformaldehyde (24 h) for hematoxylin-eosin staining (HE staining), Masson staining and apoptotic cell detection. Part of tissues were fixed with 2.5% glutaraldehyde for the observation of ultrastructure, and part of the tissues were placed in a cryotube and stored in liquid nitrogen for the determination of mRNA and related protein expression levels.

### HE staining

The myocardial tissues were fixed in 4% paraformaldehyde (24 h). The sample was dewaxed by conventional method and stained with HE staining. The sections were stained in hematoxylin and eosin staining solution (10 min), and differentiated with 1% hydrochloric acid alcohol, treated with 2% sodium bicarbonate for blue returning (10 s), and stained with eosin solution for 3 min, followed by gradient alcohol dehydration, xylene clearance, and then neutral resin sealing. Finally, the tissues were dried for 72 h, and photographed.

### Transmission electron microscopy (TEM) detection

The myocardial tissues of about 1 mm^3^ were fixed by 2.5% glutaraldehyde fixative and 1% citric acid, dehydrated with ethanol, embedded in epoxy resin Epon812, and sliced with an ultrathin slicer (Olympus, Tokyo, Japan). After staining with 3% uranyl acetate and lead citrate, the ultrastructural changes of cardiomyocytes were observed by a TEM (Hitachi, Tokyo, Japan).

### Masson staining

The myocardial tissues were fixed in 4% paraformaldehyde (24 h), sliced by conventional methods, and dewaxed to water. Tissue sections were dewaxed with xylene (3 times, 5 min/time) and soaked in gradient alcohol (100%, 90%, 85%, 75%, 5 min per time). The sections were subjected to Masson staining according to the instructions of Masson Kit (Service Biological Technology, Wuhan, China) to observe changes in myocardial fibrosis under a light microscope (Olympus, Tokyo, Japan). Image analysis was performed by using Image-Pro Plus software.

### TdT-mediated dUTP Nick-End Labeling assay (TUNEL) assay

The myocardial tissues were fixed in 4% paraformaldehyde (24 h), and then sliced into wax blocks. The cardiomyocyte apoptosis was measured by TUNEL kit (Hoffmann-La Roche Ltd., Basel, Switzerland) using a laser scanning confocal microscope (FV1000, Olympus, Tokyo, Japan). The TUNEL-positive apoptotic cardiomyocytes showed a dark brownish nucleus and a complete outline of cardiomyocytes. Determination of apoptotic index (AI): 3 sections were observed in each group, 5 non-overlapping fields were taken from each section, the number of apoptotic cells was counted. The number of apoptotic cells/total number of cells was counted.

### Reverse transcription quantitative polymerase chain reaction (RT-qPCR)

Total RNA in the myocardial tissues of each group was extracted by using an RNA extraction kit (Invitrogen, Carlsbad, California, USA). The miR-93, LIMK1, RhoA, ROCK1, U6 and glyceraldehyde phosphate dehydrogenase (GAPDH) primers were designed by Invitrogen (Carlsbad, CA, USA) (Table 1), the the internal reference of miR-93 was U6, and the internal reference of LIMK1, RhoA and ROCK1 was GAPDH. The RNA was then reversed to obtain cDNA according to the PrimeScript RT kit instructions. RT-qPCR was performed by SYBR Green PCR Master Mix kit (Roche, Arizona, USA). The relative transcription levels of the target genes were calculated by 2^-ΔΔCt^ method. The experiment was repeated three times and the final data was averaged.

**Table 1:** Primer sequence.

**Gene**	**Sequence**
miR-93	F: 5′- AGTCTCTGGCTGACTACATCACAG -3′
	R: 5′- CTACTCACAAAACAGGAGTGGAATC -3′
LIMK1	F: 5′- GGGGCATCATCA AGAGCA-3′
	R: 5′- CCAGGCAGTTGTGGGAGT-3′
RhoA	F: 5′- TCGGAATGATGAGCACACAA-3′
	R: 5′- GCTTCACAAGATGAGGCAC-3′
ROCK1	F: 5′- GTGATGGCTATTATGGACG-3′
	R: 5′- AGGAAGGCACAAATGAGAT-3′
U6	F: 5′CTCGCTTCGGCAGCACATATACT3′
	R: 5′ACGCTTCACGAATTTGCGTGTC3′
GAPDH	F: 5′- ACGGCAAGTTCAACGGCACAG -3′
	R: 5′- GACGCCAGTAGACTCCACGACA -3′

Note: F, forward; R, reverse; LIMK1, LIM domain kinase 1; GAPDH, glyceraldehyde-3-phosphate dehydrogenase.

### Western blot assay

Myocardial tissue homogenate was performed referring to the bicinchoninic acid kit (Beijing Apply Gene Technology Co., Ltd., Beijing, China) for the determination of total protein content. After denaturation, the proteins were mixed well with the loading buffer (30 μg), and boiled at 95°C (10 min). Then, 10% polyacrylamide gel electrophoresis was used, and transferred onto polyvinylidene fluoride (PVDF) membrane, after that blocked with 5% skim milk in Tris-buffered saline Tween-20 (TBST). Primary antibody against LIMK1, TNF-α, Cleaved caspase-3, Bax, Bcl-2 and GAPDH (1:1000, Cell Signaling Technology, Beverly, MA, USA), RhoA, ROCK1 (1:1000, Santa Cruz Biotechnology, Santa Cruz, CA, USA), IL-1β (1:500, Proteintech, Chicago, Illinois, USA), and IL-6 (1:1000, Abcam, Cambridge, MA, USA) were added and incubated overnight (4°C), then rinsed with TBST solution (3 times/5 min), and incubated for 1 h with the corresponding secondary antibody (Cell Signaling Technology, Beverly, MA, USA). After rinsing, enhanced chemiluminescence coloring solution was added for color development, and GAPDH was used as an internal reference. The target protein band was analyzed by gray-value analysis by using Image J software and Bio-Rad Image Lab system imaging analysis (Bio-rad, California, USA). The experiment was repeated three times and the data was averaged.

### Bioinformatics analysis and luciferase activity assay

The bioinformatics software (http://www.targetscan.org) was involved to predict the targeting relationship between miR-93 and LIMK1 and the binding site of miR-93 to LIMK1 3′-untranslated region (3′UTR). The LIMK1 3'UTR promoter region sequence containing the miR-93 binding site was synthesized, and a LIMK1 3'UTR wild type (WT) plasmid (LIMK1 3'UTR-WT) was constructed. Based on this plasmid, a binding site was mutated to construct a LIMK1 3'UTR mutant (MUT) plasmid (LIMK1 3'UTR-MUT). This procedure was carried out according to the steps of the purchased plasmid extraction kit (Promega, Madison, Wisconsin, USA). Logarithmically grown 293T cells were seeded in 96-well plates and transfected at a cell density of approximately 70% according to the instructions of Lipofectamine 2000 (Invitrogen, Carlsbad, CA, USA). LIMK1-3'UTR-WT and LIMK1-3'UTR-MUT plasmids together with miR-93 mimics and mimics NC plasmids were mixed and transfected into 293T cells. After 48 h of co-transfection, the cells were collected and lysed, and luciferase activity was measured using a luciferase assay kit (BioVision, San Francisco, CA, USA), and each set was repeated three times.

### Statistical analysis

All the data were processed by SPSS 22.0 statistical software (IBM Corp. Armonk, NY, USA). The measurement data were showed in the form of mean ± standard deviation. The measurement data obeying normal distribution between two groups was conducted by the *t* test and one-way analysis of variance (ANOVA) was used for comparison among various groups. The lest significant difference t test (LSD-*t*) method was used for pairwise comparison after ANOVA. *P* < 0.05 meant the difference was statistically valuable.

### Ethical statement

The study was agreed by the Ethics Committee of Minhang Hospital, Fudan University and informed written consent was obtained from all patients. All animal experiments were in accordance with the Guide for the Care and Use of Laboratory Animal by International Committees.
